# Desensitizing the autonomic nervous system to mitigate anti-GD2 monoclonal antibody side effects

**DOI:** 10.3389/fonc.2024.1380917

**Published:** 2024-05-15

**Authors:** Jaume Mora, Alejandra Climent, Mònica Roldán, Marta Cecilia Flores, Amalia Varo, Sara Perez-Jaume, Cristina Jou, Mónica S. Celma, Juan José Lazaro, Irene Cheung, Alicia Castañeda, Maite Gorostegui, Eva Rodriguez, Saray Chamorro, Juan Pablo Muñoz, Nai-Kong Cheung

**Affiliations:** ^1^ Pediatric Cancer Center Barcelona (PCCB), Hospital Sant Joan de Déu, Barcelona, Spain; ^2^ Department of Neurophysiology, Hospital Sant Joan de Déu, Barcelona, Spain; ^3^ Department of Genetics, Hospital Sant Joan de Déu, Barcelona, Spain; ^4^ Department of Pathology, Hospital Sant Joan de Déu, Barcelona, Spain; ^5^ Department of Pharmacy, Hospital Sant Joan de Déu, Barcelona, Spain; ^6^ Department of Anesthesiology, Hospital Sant Joan de Déu, Barcelona, Spain; ^7^ Department of Pediatrics, Memorial Sloan-Kettering Cancer Center (MSK), New York, NY, United States

**Keywords:** anti-GD2 immunotherapy, autonomic nervous system, visceral pain, neuroblastoma, infusion protocol

## Abstract

**Background:**

Anti-GD2 monoclonal antibodies (mAbs) have shown to improve the overall survival of patients with high-risk neuroblastoma (HR-NB). Serious adverse events (AEs), including pain, within hours of antibody infusion, have limited the development of these therapies. In this study, we provide evidence of Autonomic Nervous System (ANS) activation as the mechanism to explain the main side effects of anti-GD2 mAbs.

**Methods:**

Through confocal microscopy and computational super-resolution microscopy experiments we explored GD2 expression in postnatal nerves of infants. In patients we assessed the ANS using the Sympathetic Skin Response (SSR) test. To exploit tachyphylaxis, a novel infusion protocol (the Step-Up) was mathematically modelled and tested.

**Results:**

Through confocal microscopy, GD2 expression is clearly visible in the perineurium surrounding the nuclei of nerve cells. By computational super-resolution microscopy experiments we showed the selective expression of GD2 on the cell membranes of human Schwann cells in peripheral nerves (PNs) significantly lower than on NB. In patients, changes in the SSR were observed 4 minutes into the anti-GD2 mAb naxitamab infusion. SSR latency quickly shortened followed by gradual decrease in the amplitude before disappearance. SSR response did not recover for 24 hours consistent with tachyphylaxis and absence of side effects in the clinic. The Step-Up protocol dissociated on-target off-tumor side effects while maintaining serum drug exposure.

**Conclusion:**

We provide first evidence of the ANS as the principal non-tumor target of anti-GD2 mAbs in humans. We describe the development and modeling of the Step-Up protocol exploiting the tachyphylaxis phenomenon we demonstrate in patients using the SSR test.

## 1 Introduction

Systemic administration of anti-GD2 monoclonal antibodies (mAbs) in humans causes pain. Pain is rapid, severe, diffuse, and mostly visceral. This serious side effect is universal with all anti-GD2 mAbs since its first report in 1987 (1). The pain remains a major hurdle for anti-GD2 therapy development limiting combination strategies, particularly among adolescents and young adults. Besides pain, anti-GD2 mAbs commonly cause hypo/hypertension, urticaria, pyrexia, laryngospasm, bronchospasm, tachycardia, cough, vomiting, and nausea (2, 3). Although manageable, even in the outpatient setting, the emotional toll on patients, their parents, and the health care professionals, can be debilitating (4).

The pathogenesis of the pain side effect is unclear. In the rat model the allodynia produced by anti-GD2 mAbs is assumed to be immune based through complement activation damaging non-myelinating Schwann cells that surround C fibers (5). In humans, no formal evidence has been provided on the mechanism of pain or any of the side effects caused by anti-GD2 mAbs. Indeed, the transient nature of the pain in humans is out of synchrony with the prolonged pharmacokinetics of these mAbs. Furthermore, the rapid reversibility of the side effects, rarity of long-term nerve fiber damage with patient follow-up (6), and the absence of histologic proof of damage, weakened the immune-based damage hypothesis. In fact, when complement activation from mAbs was genetically removed through K322A mutation, pain side effects persisted (7).

Gangliosides are carbohydrate-containing sphingolipids (glycosphingolipids) composed of a ceramide carrying 2–3 monosaccharides (8). The ganglioside GD2 participates in the interaction with membrane proteins and lipids to regulate cellular signaling (9), while facilitating cell–cell recognition and adhesion (10). Its function in normal cellular physiology has not been fully elucidated (11, 12).

Since the 1980s GD2 expression in normal human tissues was described as restricted to neurons, lymphocytes, mesenchymal stem cells, skin melanocytes, and peripheral nerve (PN) fibers (13, 14). Early studies clearly showed that GD2 is widely expressed in the gray matter of the human postnatal brain and spinal cord (15). In the peripheral nervous system, studies using the anti-GD2 murine IgG3 antibody 3F8 stained the dorsal root ganglia of spinal nerves, sympathetic ganglion cells, and the perineurium and epineurium of PNs (13). Svennerholm et al. identified and measured the GD2 ganglioside within the PNs, subsequently confirmed by immunocytochemistry (16). The study by Yuki N. et al. in 1997 showed that all human PNs examined with the murine anti-GD2 antibody 14G2a stained positive in the myelin sheaths whereas axons and fibroblasts were negative (17). Similar findings using the same murine antibody were reported in 2011 by Alvarez-Rueda N. et al. showing intense staining of myelin sheaths in human PNs (18). In the rat, all the evidence points to the expression of GD2 on Schwann cells and the myelin surrounding the PN fibers (but not the axons). If GD2 is expressed on the glial component of the perineurium/epineurium and not the axon, the pain side effects in rats and humans could be mediated through the activation of nerve fibers (19). The nociceptive impulses produced by anti-GD2 mAbs could result from antibody binding to Schwann cell membranes that surround peripheral (unmyelinated) nerve fibers (20).

GD2 is expressed in a wide range of human tumors (21). GD2 constitutes less than 10% of the total ganglioside content of the whole nervous system but is differentially expressed in a variety of tumors. Given its restricted expression outside the CNS, GD2 is considered a tumor-associated antigen and valuable as a primary target for cancer immunotherapy that do not penetrate the CNS (21). Indeed, the U.S. National Cancer Institute ranked GD2 in the top 12 among 75 potential targets for anti-cancer therapy in 2009 (22). In neuroblastoma, the high concentration of 5–10 million of GD2 molecules per cell (14) and the homogeneity of GD2 expression within and among tumors are exceptional (23). These unique properties provided a strong rationale for the first application of anti-GD2 mAbs in neuroblastoma.

Surprisingly, anti-GD2 therapy causes pain in all patients. The pattern of pain is unique, being highly predictable in its timing of onset, consistency, and resolution within and among patients. The clinical characteristics of anti-GD2 therapy induced pain suggested a relationship with activation of the autonomic nervous system (ANS), what is known as “visceral pain”. Typical infusion (30–60 minutes) of the anti-GD2 mAb naxitamab goes as follows in the clinic (4): upon 5–7 minutes of naxitamab infusion, pain begins most commonly in the abdomen, reported as vague, diffuse, sometimes accompanied by nausea (rarely vomiting), which rapidly extends to the neck, extremities, back, and soon the “whole body”, but never to the skin, and is not accompanied by motor neurological deficits and rarely sensory changes. Pain starts when the serum level reaches 5 µg/ml (24). By 20 µg/ml pain crescendo in minutes. Heart rates and respiratory rates increase while oximetry remains normal. Patient usually requires multiple doses of an opioid, despite which pain persists, and increases until it peaks after 12–15 minutes. At that point, infusion is often slowed or interrupted by the caretaker when confronted with grade 3 (G3) arterial hypotension. Fluid boluses are usually administered because of refractory hypotension. After holding anti-GD2 infusion, when symptoms resolve and blood pressure recovered, infusion is restarted to reach the standard infusion rate (3 mg/kg over 30–60 minutes). Within minutes bronchospasm can occur requiring salbutamol nebulization and oxygen supplementation. Heart rate now increases significantly (120–140 per min). When naxitamab infusion is completed ~50 minutes after initiation, vague and diffuse pain usually necessitates rescue doses of analgesics. Serum hu3F8 at the end of infusion peak at a mean level of above 50 +/- 9 (range 27–85) µg/ml. By one hour after the end of naxitamab infusion, patient has usually recovered, typically sleeping, without need of supplemental oxygen. Heart rate tends to remain high (130–155 per min) even though there is usually no evidence of pain or distress. By two hours after mAb infusion, patient wakes up, and following a clearance examination, is discharged home, ambulatory, with mostly normal vital signs except for increased heart rate but no pain. By then serum level is expected to be ~40 µg/ml and by 6 hours 18 +/- 4 µg/ml (24), much higher than the threshold required for pain at the first encounter. However, patient remains pain-free with only minor discomfort through the next 48 hours before the next dose of naxitamab, when the trough level is still quite high at around 18 µg/ml.

The ANS differs from the somatic motor system in many ways. Only one neuron is required to transmit somatic motor impulses, but autonomic impulses (efferent branch) are transmitted by a chain of at least two neurons. These two neurons have different embryonic origins, with the second neuron originating from the neural crest (NC). Preganglionic fibers are myelinated and use acetylcholine as neurotransmitters. Postganglionic nerve fibers are smaller, unmyelinated, and use norepinephrine as neurotransmitter. The exception are sweat glands, which use cholinergic nerves (25). Electro physiologically, the peripheral nervous system is classified into different types of nerves, based on diameter, myelin sheet, and conduction velocity (26). Myelinated fibers show faster conduction velocities compared to smaller unmyelinated C-fibers (27). Most unmyelinated fibers in the human body belong to the ANS.

The sympathetic sudomotor skin response (SSRs) is defined as a transient change of the skin electrical potential, either spontaneous or evoked (28, 29). SSR is used routinely in medical practice to evaluate pre- and post-ganglionic sympathetic activity by measuring the change in voltage originating on the surface of the skin, usually, after electrical stimulation. The electrical stimulation is passed on sensory myelinated fibers to the spinal cord up to sympathetic neurons and ascend to superior circuits in the CNS (28). The efferent segment of this response is elicited by the hypothalamus, descend throughout lateral columns in the spinal cord to end at the preganglionic sympathetic neurons. In the sympathetic ganglia, peripheral post-ganglionic sympathetic Sudomotor fibers are originated. These are amyelinic fibers, C-type that join the PNs to arrive to the sweat eccrine glands located at the epidermis.

The observation in the rat model of anti-GD2 induced pain where allodynia occurred along with an ectopic activity in afferent C-fibers (19) is informative. Given the fact that most C-fibers in the human body are found in the vagus nerve (VN) (27), the effect of anti-GD2 mAbs on the VN could be responsible for many of the side effects encountered in the clinic. In this study, we provide first evidence of the ANS as the principal non-tumor target of anti-GD2 mAbs in humans. Building on these pathophysiological findings, we developed the Step-Up infusion protocol whereby modifying the pharmacodynamics of naxitamab is able to significantly reduce the intensity of side effects.

## 2 Results

### 2.1 Anti-GD2 mAb naxitamab in humans cause ANS activation

When rats were treated with anti-GD2 antibodies, resulting allodynia is complement-dependent and measurable (5). As the pain behavior was blocked by systemic pre-treatment with a complement C5 antagonist (30), the assumption has been that the nociceptive actions produced by anti-GD2 therapy occur downstream of an antibody-antigen interaction on the non-myelinating Schwann cells that surround C-fibers. In humans, removing complement activation from the anti-GD2 mAb did not mitigate pain side effects (7). Furthermore, the pain pattern in humans suggests an alternative pathophysiology. Rather than an immunological complement mediated damage, we postulate a reversible electro physiological discharge throughout the ANS, mainly the VN, to explain the side effects of anti-GD2 mAbs in humans.

**Figure 1:**
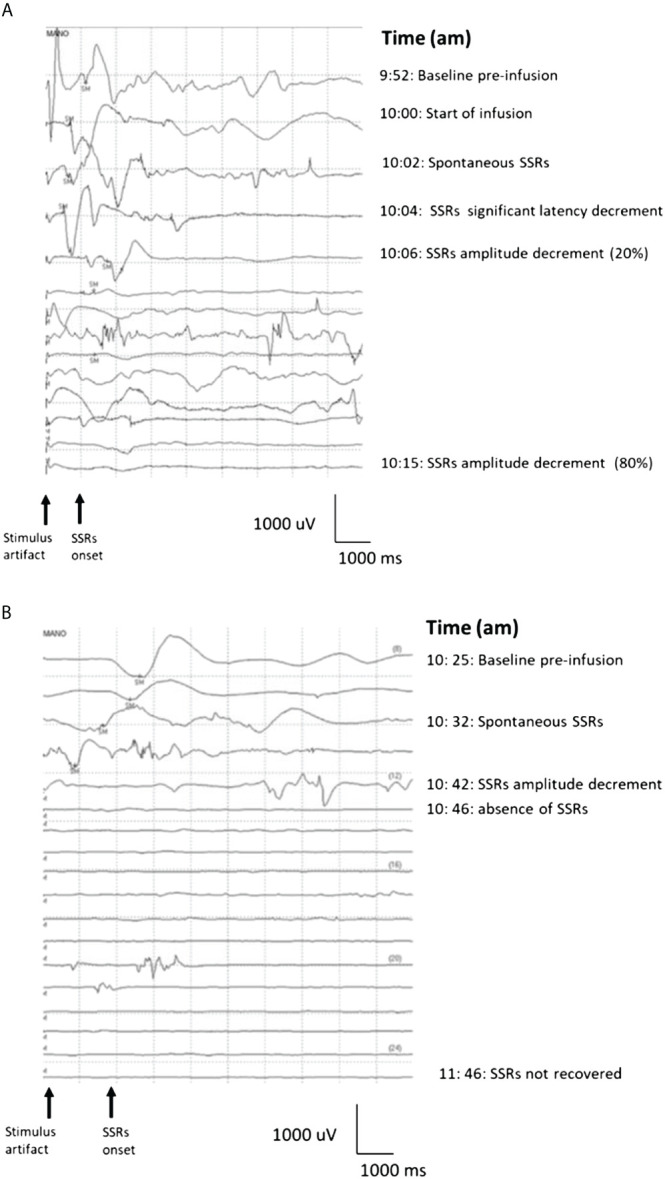
**(A)**Sympathetic Skin Response Test waterfall in real time for a neuroblastoma patient treated with naxitamab at day one of first cycle. SM= electric stimuli. Baseline (9:52 am) was recorded before starting infusion at 10 am. Two minutes after the start of naxitamab infusion spontaneous SSRs occurred with latency decreased. The SSRs 6 minutes after the infusion began show a significant (20%) amplitude decrement that was not recovered one hour after the infusion was delivered. By minute 15 post infusion start, almost complete amplitude decrement (>80%) was recorded. **(B)** Day 5 of the cycle (3^rd^ dose of naxitamab within the cycle) SSRs waterfall from same patient. Baseline was recorded before starting the infusion (10:25 am). After 5 minutes of infusion SSRs amplitude started to decrease along a latency decrement. This change abruptly evolved into complete disappearance of the signal 12 minutes after the infusion start. Similar tachyphylaxis patterns were reproduced for days 3 and 5 among the 10 NB patients studied.

To test our hypothesis, we first monitored the ANS response during the anti-GD2 mAb naxitamab infusion in high-risk neuroblastoma (HR-NB) patients. We adapted the SSR test to evaluate the efferent unmyelinated axon function of the ANS in patients treated with naxitamab. **Figure 1A** shows the typical SSR register of a HR-NB patient at baseline using palm electrodes (active in the palm and a dorsal one as reference) after stimulation of the sympathetic conduction by a forearm electrode (3 cm apart cathode and anode). A baseline SSR curve is obtained before naxitamab treatment with normal latency and amplitude on day one of a treatment cycle. In every patient studied, after 3 to 5 minutes from the beginning of naxitamab infusion, a change in the SSR response was noted. First, the latency of the response was abruptly shortened, accompanied by gradual decrease in the amplitude over time before its complete disappearance (**Figure 1A**). SSR response did not recover at one hour after the end of infusion, at which point the SSR study was stopped before patient discharge from the clinic. SSR study was carried out in 10 unique patients over several naxitamab cycles and on either day 1, 3 or 5 of each treatment cycle. The blunting of the SSR response was consistent and reproducible across patients, irrespective of day of the naxitamab cycle and the treatment cycle # (**Figure 1A**).

### 2.2 GD2 as target for electro-physiological activation

The antigen-binding domain of the anti-GD2 mAbs recognize disialoganglioside GD2, while the Fc domain recruits effector white blood cells to promote tumor cell lysis via antibody-dependent, cell-mediated cytotoxicity (ADCC) or complement mediated cytotoxicity (CMC). Killing efficiency depends on the affinity of the anti-GD2 antibodies and on target density on tumor cells (31), providing neuroblastoma selectivity because of its abundance over normal tissues. Furthermore, initial tests by ELISA and immunofluorescence did not show nonspecific staining of peripheral blood or bone marrow derived mononuclear cells.

**Figure 2:**
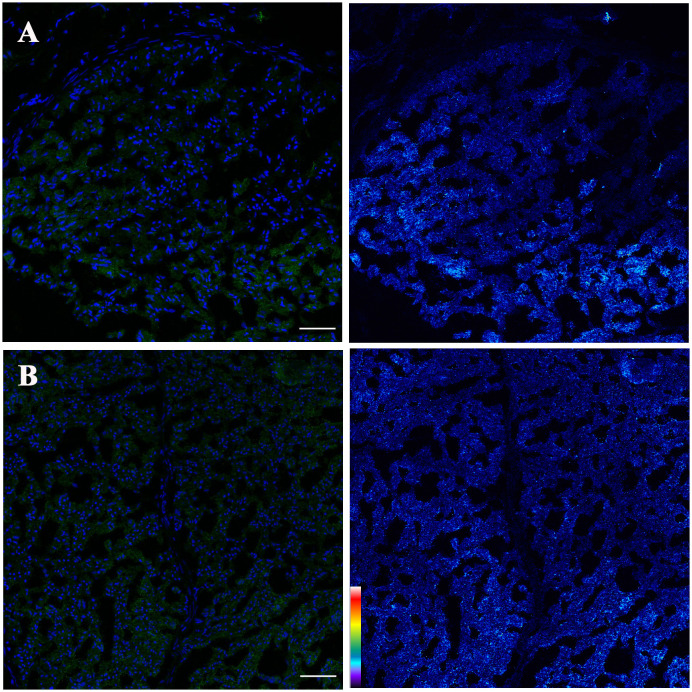
Confocal projections of GD2 expression (left: blue= nuclei, green= GD2; right: pseudo color image corresponding with fluorescence intensity GD2) of **(A)** Phrenic nerve and **(B)** Vagus nerve. GD2 is localized surrounding the nerve cells (Left panels). Pseudo color palette (Right panels) has been added to visualize the different degrees of intensity of GD2. Warm colors such as white and red represent maximum intensities, whereas cold colors like blue are representative of low intensities. Low signal intensity is observed. The pseudo color scale is shown at the bottom left. Scale bar = 50 μm.

**Figure 3:**
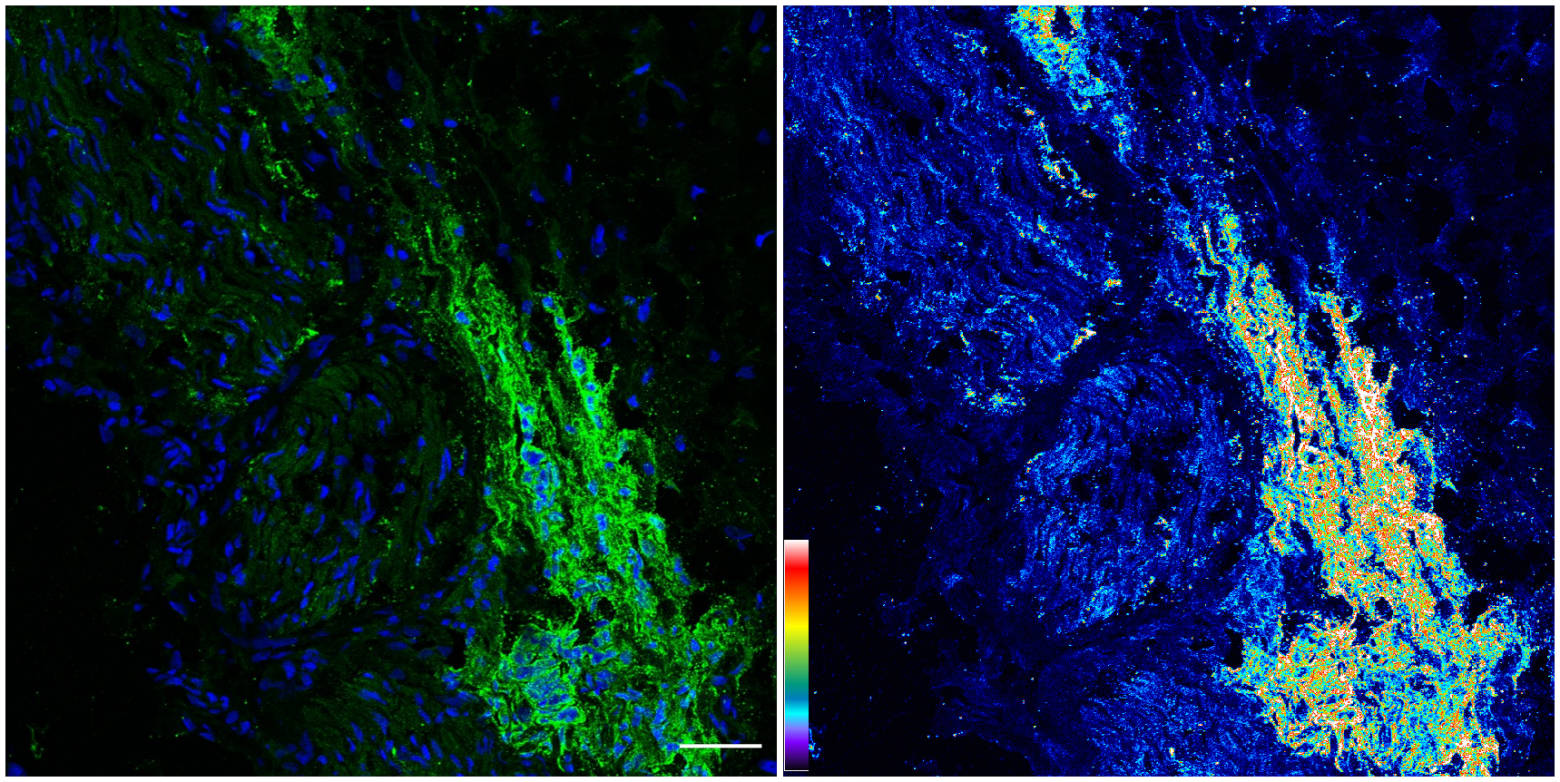
Confocal image of GD2 expression from neuroblastoma tumor: Same image in the Left panel (original image with blue= nuclei, green= GD2) and Right panel (corresponding pseudo color image with fluorescence intensity for GD2). An increase in fluorescence intensity was observed in pseudo color image (Right) versus the expression in Phrenic and Vagus nerves. The pseudo color scale is shown at the bottom left. Scale bar = 50 μm.

**Figure 4:**
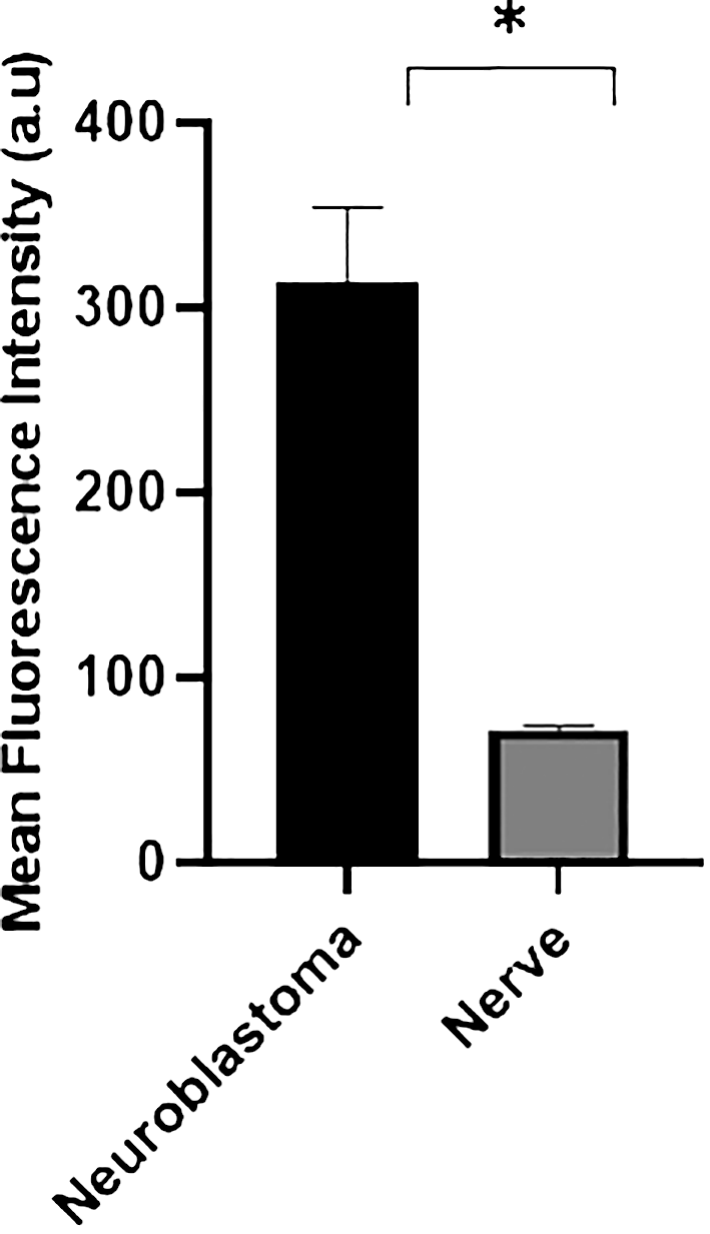
Quantitative confocal imaging. Fluorescence intensity analysis was also quantified in neuroblastoma (n=3) and nerve samples from 3 different infants at autopsy. Data are presented as mean ± SD (n = 3). Student’s t test was used to compare the data (*p < 0.05).

We revisited the issue of GD2 expression in postnatal samples and specifically in the PNs of neonates at autopsies. **Figure 2** shows the immunofluorescence analysis of the phrenic nerve (**Figure 2A**) and the VN (**Figure 2B**) from 3 different infants at autopsy through confocal microscopy. In these images, GD2 expression is clearly visible (in green) in the perineurium surrounding the nuclei of nerve cells. The GD2 expression level was quantified at 72.01 ± 2.62 arbitrary units (AU) in both the phrenic nerve and the VN, whereas it measured at 314 ± 40.52 AU in neuroblastoma cells. It is worth noting that this level of expression in normal nerves is undetectable using conventional fluorescence microscopy. **Figure 3** is a confocal microscopic image of a phrenic nerve next to a surgical neuroblastoma sample. By quantitative imaging, the expression of GD2 in nerves of the peripheral nervous system (including the ANS) in infants (n=3) is significantly lower (Student’s t test p < 0.05) than neuroblastoma tumor cells (**Figure 4**).

**Figure 5:**
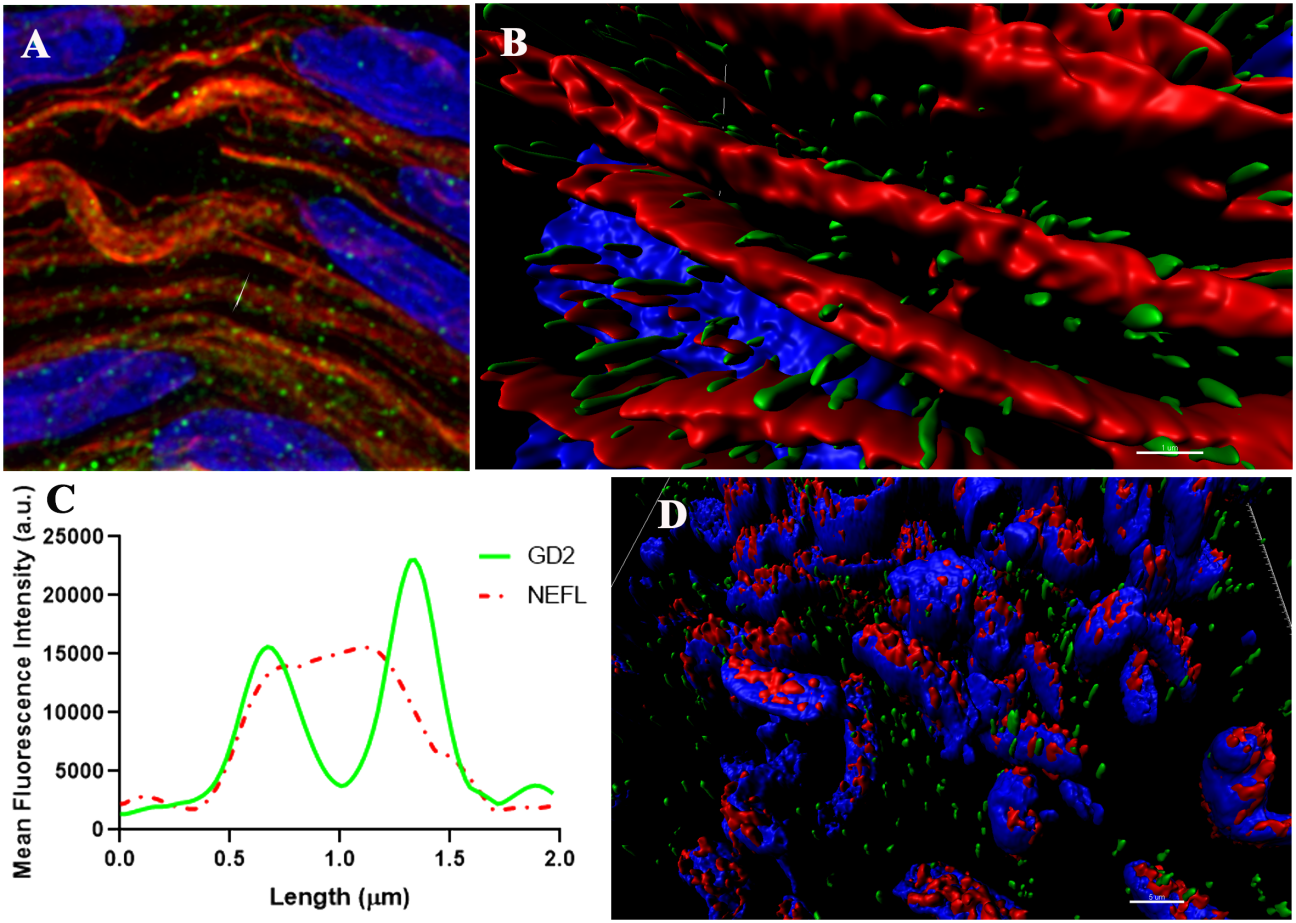
Super-resolution images of the Vagus nerve (blue = nuclei, green = GD2, red=NEFL or SOX10). **(A, B)** representative 3D image illustrating the distribution of GD2 around NEFL (neurofilament). **(C)** the corresponding intensity profile of GD2 (green channel) and NEFL (red channel) from **(A, B)** along an ideal straight line (dotted red) crossing the event. Scale bar: 1 µm. (AU, Arbitrary Units). **(D)** representative 3D image showing the distribution of GD2 and SOX10. Scale bar= 5 µm.

The GD2 expression in the rat model is reported in the Schwann cells surrounding unmyelinated nerves (5). In humans, GD2 expression in the PN has not been extensively reported. Computational super-resolution microscopy of GD2 and neurofilament in PNs is shown in **Figures 5A–C**. GD2 was found in the cell membranes of SOX10 positive nucleated cells, a well-known marker of Schwann cells surrounding the nerve fibers (**Figure 5D**). Importantly, GD2 expression was found at similar levels in both myelinated phrenic nerve and unmyelinated VN.

### 2.3 Pharmacokinetics and pharmacodynamics. a new infusion regimen

Study of the pharmacokinetics (PK) of anti-GD2 mAb ch14.18 in 14 neuroblastoma patients suggested that antibody clearance was fourfold higher in younger children, and appeared to be age dependent (32). However, even in the same age group, weights could vary 2.5 to 3 folds in children (33). Various models have been proposed to account for the differences in drug clearance based on weight and age (34–36). For naxitamab, AUC was strongly correlated with patient body weight at any dosage level (24). Based on the PK, naxitamab threshold for severe pain starts at 20 µg/ml, which could be achieved beyond 1 mg/kg dose levels. However, as the dose increases to 3.6 mg/kg and the AUC rises, pain intensity does not substantially change (24). Previous studies of other anti-GD2 antibodies also failed to show a clear relationship between mAb dose and pain (37, 38). In addition, pain intensity was independent of infusion-time at these antibody dose levels.

Another universal observation of anti-GD2 mAbs induced pain is the pattern of diminishing intensity with subsequent infusions after the first day of each cycle (39). Even though the same dose (3 mg/kg) of naxitamab is given on each day of the cycle, the side effects are much worse, with intense pain and ANS reactivity, on the first day (usually Monday), compared to the second day (usually Wednesday), or the third day (usually Friday). This pattern can be explained by tachyphylaxis or nerve desensitization if neuronal potentials underscored the side effects. To assess tachyphylaxis of the ANS the SSR test was repeated on days 3 (second dose of naxitamab within a cycle) and 5 (third dose of naxitamab within a cycle) (**Figure 1B**) for the same patient in **Figure 1A**. At baseline, before infusion, a lower amplitude wave was seen and after infusion started, a faster loss of the signal observed. A clear pattern of tachyphylaxis is demonstrated whereby the sympathetic nerves are increasingly depolarized over sequential doses coinciding with the attenuation of side effects observed over repeated doses.

**Figure 6:**
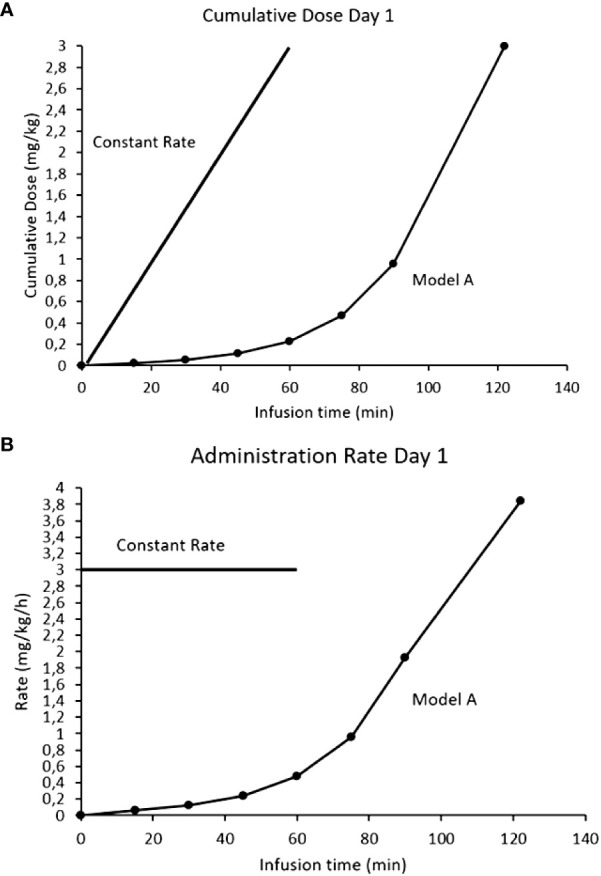
Comparison models of infusion rates for naxitamab infusion of Day1: constant rate (current model) vs Step-Up model. **(A)** Infusion rates over time. **(B)** Cumulative dose over infusion time. In the Step-Up model 16% of the dose is administered the first 75 minutes (below the 1 mg/kg at which the 20 ug/ml level is reached) while the ANS is being depolarized. Infusion target dose of 3 mg/kg is completed during the remaining 45 minutes at a much faster administration rate in the repolarization (refractory) phase.

**Figure 7:**
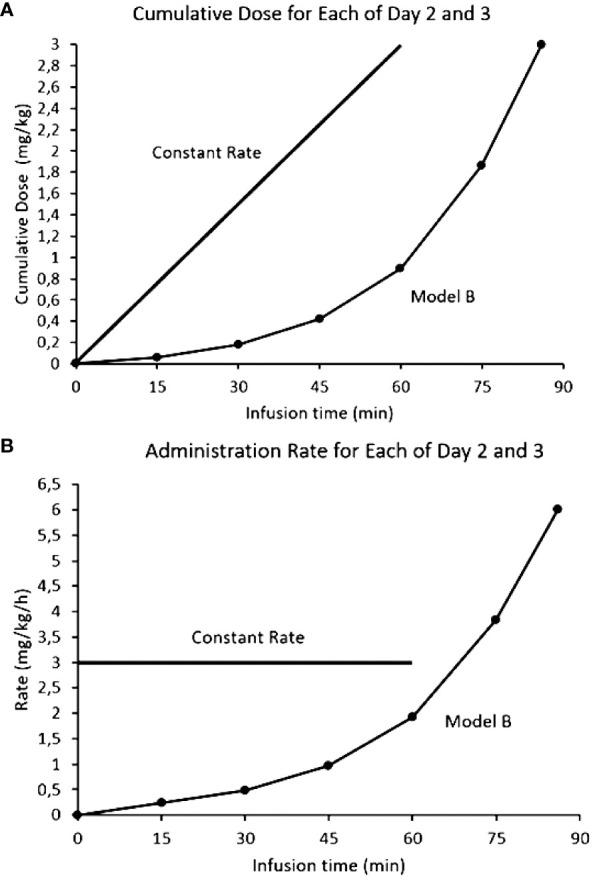
Comparison models of infusion rates for naxitamab infusion of Days 2 and 3: constant rate (current model) vs Step-Up model. **(A)** Infusion rates over time. **(B)** Cumulative dose over infusion time. In the Step-Up model 16% of the dose is administered the first 45 minutes (below the 1 mg/kg at which the 20 ug/ml level is reached) while the ANS is being depolarized. Infusion target dose of 3 mg/kg is completed during the remaining 45 minutes at a much faster administration rate in the repolarization (refractory) phase.

If activation of the ANS is responsible for the side effects (the neurotransmitter response can be desensitized) and there is a mAb threshold for pain, a novel approach to mitigate the side effects could be through exploiting the induced tachyphylaxis by dissociating on-target off-tumor effects from tumor drug exposure (or area under the curve, AUC). If acute depolarization of all the activatable nerves in the ANS is what generates clinically significant toxicities, we hypothesized that subsets of ANS nerves with increasing activation thresholds could be desensitized in a step wise fashion. By activating and desensitizing these small ANS subsets using a stepwise mAb dose escalation, the entire ANS could become finally depolarized to allow large antibody doses to be infused with blunted side effects. The desensitization protocol (the Step-Up) for naxitamab was modelled after the neurophysiological data obtained from patients using quantile increases of the infusion dose over 75 minutes (16% of the total dose) completing the Step-Up to a final cumulative target dose of 3 mg/kg over the remaining 45 minutes (hyperpolarized phase) for a total infusion time of 2h for day one of the cycle. **Figure 6** depicts the mathematical representation of the naxitamab infusion rate over time (**Figure 6A**) and the cumulative dose over infusion time (**Figure 6B**) in the Step-Up model for Day 1 of the cycle. For subsequent dosing of the cycle (naxitamab infusion days 2 and 3) and following the desensitization observed in the neurophysiological data that correlated with the reduction in side effects, a faster 90 minutes total Step-Up program is modelled in **Figure 7**. The 16% of the dose is administered the first 45 minutes while the ANS is being depolarized. Infusion target dose of 3 mg/kg is completed during the remaining 45 minutes at an accelerated rate in the repolarization (refractory) phase.

### 2.4 Toxicity profile of the step-up protocol

The Step-up infusion protocol was initially tested in a total of 42 patients and recently reported (40). The G3 adverse events occurrence rate was significantly reduced from 8.1% (23 out of 284 infusions) with standard administration to 2.5% (5 out of 202 infusions) with the Step-Up regimen (p=0.037). The odds of a G3 adverse event occurring were reduced by 70% with the Step-Up protocol (40). Using the Step-up infusion procedure, naxitamab serum levels were collected before antibody (Ctrough) and after its completion (Cmax) for 12 patients over their multiple treatment cycles as previously reported (24). Drug exposure did not appear to be negatively affected by the Step-Up infusion procedure (40). Since the Step-Up was implemented, over 150 patients have been infused and no CTCAE grade 4 toxicities have been observed and thus all patients have been able to complete planned infusions.

Importantly, the new infusion protocol permitted a decrease in the level of monitoring required. With the standard infusion protocol, two nurses had to be present at the patient’s bedside together with the infusion physician (4). Usually one nurse is responsible for medication, whereas the other nurse monitors vital signs and completes charting. The third nurse remains outside the infusion suite and is responsible for administering chemotherapy (as appropriate), post-naxitamab infusion patient monitoring/follow-up, and providing premedication to patients awaiting naxitamab infusion (4). With the Step-Up protocol, there is no more need for an MD to stay at the bedside and only one bedside nurse is required given the decrease in the intensity of infusion reactions.

## 3 Discussion

Every organ of the human body is monitored by the ANS while it tries to keep “sympathy” (Galen) between functional systems (41). Although close to viscera in general, the ANS innervation can be found in salivary glands, blood vessels, smooth muscle, and others (42). The autonomic nervous efferent branch is transmitted by two neurons with different embryonic origins. The second neuron originates from the NC, of particular relevance here because neuroblastoma appears to recapitulate the development of differentiating sympathetic neurons of the ANS (43). Such common embryonic origins might explain the GD2 expression found in ANS nerves as well as the exquisite affinity of the anti-GD2 mAbs for the ANS as shown in this study. We provide quantitative evidence of GD2 expression in the nerves of the ANS of the post-natal human body as well as evidence of neurophysiological activation of the sympathetic nervous system by the anti-GD2 mAb naxitamab. According to our results, most if not all of the off-tumor side effects caused by anti-GD2 mAbs are directly or indirectly related to the activation of the ANS. Consequently, modulating the activity of the ANS we were able to temperate the side effects produced by anti-GD2 therapy.

The VN is the longest nerve in the body. The VN is a mixed nerve containing different types of fibers including large myelinated efferents providing somatic innervation to laryngeal muscles, whereas smaller fibers provide parasympathetic, cholinergic, innervation to visceral organs (44). Large myelinated afferents provide somatic sensation to the pharyngeal and laryngeal mucosa, whereas smaller fibers provide visceral mechanical and chemical sensation to lungs and airways, vessels and the gastrointestinal tract. Unmyelinated (C-type) efferents mediate sympathetic innervation of visceral organs (44) whereas C-type afferents provide nociception, temperature and chemical sensation (45, 46). The VN is the main component of the cranial parasympathetic ANS. The other parasympathetic component of the ANS is the sacral parasympathetic nucleus (S2–S4) at the origin of the pelvic nerves that provide innervation to the pelvic organs such as the bladder, genitals, and left recto-colon. The VN ensures a bidirectional communication between the CNS and the viscera, in particular the digestive tract (47). The functioning of the viscera is usually not perceived but can, under certain pathological conditions (i.e. intensity of signal) become perceived as painful. Vagal afferents (unmyelinated) inform the CNS, usually unconsciously, of the functional state of the viscera, mainly the gastrointestinal tract. Vagal afferents originate from NC derived neuroblastic cells located in the different layers of the viscera wall and travel (unmyelinated) through the VN to the medulla (48, 49). CNS modulation of the functioning of the ANS occurs via descending pathways projecting onto sympathetic pre-ganglionic neurons in the spinal cord and onto the VN. Given the importance of the VN within the ANS, most symptoms of patients treated with anti-GD2 mAbs can be related to an activation of the parasympathetic component of the ANS: cough (bronchospasm and laryngospasm); hypoxemia (lung vascular shunting); abdominal visceral pain (splanchnic nociception); nausea-vomiting-diarrhea (enteric viscero-motor activation); and most importantly hypotension (vasodilation).

Most organs of the body are innervated by efferent autonomic nerve fibers from both the sympathetic and parasympathetic ANS. The cholinergic/noradrenergic balance is apparently a unique feature of the primate ANS (50). Both systems usually compensate one to another in response to stimuli. For anti-GD2 therapy, however, both systems are activated at the same time, which makes interpretation of the symptoms quite distressing for clinicians. Patients treated with naxitamab show hypotension as well as persistent tachycardia, which is not compensatory. Hypotension usually resolves with volume replacement for the splanchnic vasodilation but the tachycardia persists regardless of the blood pressure outcome. Tachycardia is also unrelated to pain. Patients on anti-GD2 immunotherapy persist with sympathetic activation (tachycardia) after the pain is over.

The Sudomotor and cardiovascular components of the ANS control thermoregulation of the human body. Two separate efferent pathways regulate temperature control (51). These pathways are somatic motor fibers mediating an increase in body temperature by inducing muscle shivering as well as sympathetic fibers regulating blood vessel and Sudomotor function. Postganglionic control of cutaneous sweat glands is mediated by axons of these neurons which innervate the skin as unmyelinated C-fibers. The SSR test measures the unmyelinated C-fiber conduction of the postganglionic sympathetic Sudomotor component of the ANS (28, 29). The SSR tests conclusively and reproducibly showed consistent activation of the sympathetic nervous system upon naxitamab infusion with rapid depolarization of the nerve. After 20–25 minutes, the nerve became unresponsive to electric stimuli and a hyperpolarization phase remained at least for 24 hours. Partial recovery was shown two days after the first infusion, which provided a different pattern of depolarization for days 3 and 5 of the cycle responsive for the tachyphylaxis observed in the clinic.

The clinical characteristics of anti-GD2 induced pain do not permit to qualify it as neuropathic. Neuropathic pain is an umbrella term for a series of different conditions caused by a lesion or disease of the parts of the nervous system that usually signal somatosensory information (52). Extensive clinical experience with NB patients treated for decades with anti-GD2 mAbs do not describe areas of sensory loss where the pain is manifested; do not usually describe pain as burning; do not show an increased pain over repeated stimuli (anti-GD2 doses), on the contrary, usually a decrease of pain is observed over time; and the pain most commonly disappears once therapy is completed. These characteristics clearly exclude neuropathic pain as the clinical description of anti-GD2 induced pain in humans. Some HR-NB patients receiving anti-GD2 mAbs can describe features of neuropathic pain but it should be reminded that all HR-NB patients receive neurotoxins (platinum agents, vinca alcaloids) a well-known cause of neuropathic pain. Our data suggests that anti-GD2 therapy causes visceral pain, which is perceived more diffusely than noxious cutaneous stimulation. Usually visceral afferents synapse at multiple spinal levels, which causes a diffuse localization of the initial noxious signal (53). The treatment of visceral pain is not well developed and most of the approaches used for somatic pain, including opioids, are not as effective. Our proposal of a novel infusion protocol helps decreasing the visceral pain experience of patients undergoing anti-GD2 therapy by pharmacodynamically modulating the activity of the ANS response.

In conclusion, we here provide first evidence of the ANS as the principal non-tumor target of anti-GD2 mAbs in humans. The neurophysiological activation of the ANS by anti-GD2 mAbs demonstrated in HR-NB patients using the SSR test explains many of the side effects encountered during infusion. We describe the development and modeling of the Step-Up protocol exploiting the tachyphylaxis phenomenon well described in the clinic.

## 4 Materials and methods

We report on High-Risk Neuroblastoma (HR-NB) patients treated at Hospital Sant Joan de Déu, Barcelona with naxitamab-based immunotherapy in complete remission according to previously reported experience (54). Informed written consents for treatments and tests were obtained according to HSJD institutional review board rules.

### 4.1 Anti-GD2 immunotherapy treatment

Naxitamab-based immunotherapy cycles comprised priming doses of subcutaneous GM-CSF for 5 days at 250 μg/m2/day (days -4 to 0), followed by naxitamab + subcutaneous GM-CSF for 5 days at 500 μg/m2/day (days 1–5). Standard naxitamab protocol infusion is provided over 60 minutes at 3 mg/kg/day on day 1 and over 30 minutes on days 3 and 5 for a total dose of 9 mg/kg per cycle. GM-CSF was not given if the ANC was > 20,000/μl. Treatment cycles were repeated every 4 weeks (± 1 week) for a total of 5 cycles, if patient remained in CR (54).

### 4.2 The sympathetic skin response test

Assessment of the Sympathetic Skin Responses was performed by continuous measurement of the electrodermal activity following sympathetic stimulation with a surface electromyography electrode placed on the patient’s palm or sol and a reference electrode (51). We adapted the SSR test developed for spinal cord trauma or diabetic neuropathy to our patient population.

To elicit the SSRs, electric stimulation was performed using a pair of self-adhesive disk surface electrodes 2.5 by 2.5 cm (AMBU, Ballerup, Denmark). The electrodes were placed as cathode (distal) and anode (proximal) 3 cm apart along the ventral forearm, 3 cm distal to the elbow. One single pulse stimulus with 1 millisecond duration was applied. The recording electrodes were the same to those used for stimulation. They were placed as a ventro-dorsal montage in the hand. The active electrode in the palm and the reference electrode in the back of the hand. An extra identical electrode was placed along the forearm as a ground. The response was monitored during naxitamab infusion and 1h after the infusion was delivered. Baselines were taken before starting naxitamab infusion. Once the antibody infusion started, a new signal was elicited and collected every two minutes.

### 4.3 Histology, immunostaining, confocal and super resolution imaging

The tissue blocks were cut into 3-μm thick sections and were stained with hematoxylin and eosin (HE). Light microscopy images were acquired with a DFC700T digital camera on a DM5500B light microscope using LAS X software (all by Leica Corp.). Three microns thickness sections were cut and after 20 minutes at room temperature (RT) they were fixed with paraformaldehyde 4% for 20 minutes at RT. Then were abundantly rinsed with PBS and then blocked for 90 minutes with PBS-BSA 3% at RT. After that, sections were directly incubated at RT 45 minutes with primary antibody anti-GD2 mouse IgG 1:700 (BD Pharmingen Monoclonal Clone: 14.G2). Samples were washed with PBS three times, then incubated with secondary antibody Donkey Anti-Mouse IgG H&L (Alexa Fluor^®^ 488) for 45 minutes at room temperature. The samples were also labeled by anti-NEFL (1:100, Thermo Fischer Scientific, Inc) and SOX10 (1:500, Sigma Aldrich) with the same protocol and overnight and secondary antibody Donkey Anti-Rabbit IgG H&L (Alexa Fluor^®^ 594). For counterstaining of nuclei, sections were incubated 2 minutes with 1:2000 DAPI (Thermo Fisher Scientific, Inc) and rinsed in PBS before mounting onto Fluoromount G (Thermo Fisher Scientific, Inc).

Confocal microscopy provided the ability to capture images deep within tissues and enables optical sectioning for 3D reconstructions of imaged samples. Specifically, we used a Leica TCS SP8 confocal microscopy equipped with Hybrid spectral detectors (Leica Microsystems GmbH, Mannheim, Germany). These hybrid spectral detectors with high sensitivity enhanced the signal-to-noise ratio, leading to improved and sharper detection of the GD2 target/biomarker. Images were acquired using an HC x PL APO 20x/0.75 dry. DAPI was excited with a blue diode laser (405 nm) and detected in the 425–480 nm. Anti-GD2 antibody bond to Alexa Fluor 488 was excited with an argon laser (488 nm) and detected in the 500–550 nm. Z stacks of 10 sections were acquired every 1.5 μm along with the tissue thickness. Appropriate negative controls were used to adjust confocal settings to avoid non-specific fluorescence signals. Identical confocal conditions were consistently applied for image acquisition to the set of samples to be compared in the different experiments. Maximum intensity projections were generated using LAS X software and fluorescence quantification was performed using the ImageJ software (National Institutes of Health, Bethesda, MD, USA).

For the computational super-resolution images (HyVolution mode), the HC PL APO CS2 100×/1.40 Oil objective and Hybrid detectors (HyD) were used. For Anti-GD2 and DAPI the same settings of excitation and emission that confocal were used. Anti-NEFL and Anti-SOX was excited at the 594 nm and their fluorescence emission detected at 610–785 nm. 1024 × 1024 pixel images were acquired with a pixel size of 21 nm and pinhole was set to 0.8 AU. To study GD2 distribution in three dimensions, Z stacks were acquired every 0.35 μm along the tissue thickness. Image deconvolution was performed with Huygens Professional Software v17.10.0p8 (SVI, Leiden, The Netherlands). Super-resolution images were processed using the Surpass Module in Imaris software (Bitplane).

### 4.4 Statistical analysis

A logistic mixed model (estimated using maximum likelihood) was fit using R (55) and the lme4 package (56) to analyze the relationship between the type of infusion administration (Standard vs StU) and the occurrence of a G3 or G4 adverse event. The model included individual and cycle number as random effects and type of infusion administration as a fixed effect; odds ratios were derived from this model. The Wald approximation was used to compute 95% confidence intervals and p-values. P-values under 0.05 were considered statistically significant.

Graphs were created and statistical analysis was performed using GraphPad Prism version 8.0.1 (GraphPad Software, Inc., La Jolla, CA, USA). Student´s t test was used for compare the data (*p < 0.05; **p < 0.01; ***p < 0.001).

## Data availability statement

The raw data supporting the conclusions of this article will be made available by the authors, without undue reservation.

## Ethics statement

The studies involving humans were approved by and conducted at Hospital Sant Joan de Deu, Barcelona. The studies were conducted in accordance with the local legislation and institutional requirements. Written informed consent for participation in this study was provided by the participants’ legal guardians/next of kin.

## Author contributions

JM: Conceptualization, Data curation, Formal analysis, Funding acquisition, Investigation, Project administration, Resources, Supervision, Validation, Writing – original draft, Writing – review & editing. ACl: Investigation, Visualization, Writing – review & editing. MR: Investigation, Visualization, Writing – review & editing. MF: Investigation, Writing – review & editing. AV: Investigation, Writing – review & editing. SP-J: Data curation, Methodology, Software, Validation, Visualization, Writing – review & editing. CJ: Investigation, Writing – review & editing. MC: Investigation, Methodology, Writing – review & editing. JL: Investigation, Methodology, Writing – review & editing. IC: Investigation, Methodology, Writing – review & editing. ACa: Investigation, Methodology, Writing – review & editing. MG: Investigation, Methodology, Writing – review & editing. ER: Investigation, Writing – review & editing. SC: Investigation, Methodology, Writing – review & editing. JP: Investigation, Writing – review & editing. NKC: Conceptualization, Supervision, Validation, Writing – original draft, Writing – review & editing.
